# Augmented drug combination dataset to improve the performance of machine learning models predicting synergistic anticancer effects

**DOI:** 10.1038/s41598-024-51940-9

**Published:** 2024-01-18

**Authors:** Mengmeng Liu, Gopal Srivastava, J. Ramanujam, Michal Brylinski

**Affiliations:** 1 Division of Electrical and Computer Engineering, Louisiana State University, Baton Rouge, LA 70803 USA; 2 Department of Biological Sciences, Louisiana State University, Baton Rouge, LA 70803 USA; 3 Center for Computation and Technology, Louisiana State University, Baton Rouge, LA 70803 USA

**Keywords:** Computational biology and bioinformatics, Data processing, Machine learning

## Abstract

Combination therapy has gained popularity in cancer treatment as it enhances the treatment efficacy and overcomes drug resistance. Although machine learning (ML) techniques have become an indispensable tool for discovering new drug combinations, the data on drug combination therapy currently available may be insufficient to build high-precision models. We developed a data augmentation protocol to unbiasedly scale up the existing anti-cancer drug synergy dataset. Using a new drug similarity metric, we augmented the synergy data by substituting a compound in a drug combination instance with another molecule that exhibits highly similar pharmacological effects. Using this protocol, we were able to upscale the AZ-DREAM Challenges dataset from 8798 to 6,016,697 drug combinations. Comprehensive performance evaluations show that ML models trained on the augmented data consistently achieve higher accuracy than those trained solely on the original dataset. Our data augmentation protocol provides a systematic and unbiased approach to generating more diverse and larger-scale drug combination datasets, enabling the development of more precise and effective ML models. The protocol presented in this study could serve as a foundation for future research aimed at discovering novel and effective drug combinations for cancer treatment.

## Introduction

Developing effective anticancer therapies is an important yet challenging task. Most currently available treatments employ a monotherapy, i.e., using a single drug to treat a particular disease^1,2^. Although widely used, monotherapies are known to suffer from certain problems, such as the acquired drug resistance and prominent side effects^1,3^. In contrast, combination therapies utilizing multiple pharmaceuticals to simultaneously target several biological processes generally have greater chances of overcoming these issues^4^. Not surprisingly, combination therapies against complex diseases, such as cancer, are attracting a significant attention. Nonetheless, exploring all possible drug combinations within a vast pharmacological space is a major obstacle to find those drug combinations exhibiting synergistic effects. Accurate computational methods to select the most promising therapeutic candidates for experimental testing can greatly facilitate the discovery of effective drug combinations.

Approaches utilizing machine learning (ML) are well suited to predict drug synergistic effects. Supervised learning techniques require large-scale experimental data to train models predicting effective drug combinations. These datasets differ with respect to the number of drugs and cell lines. For instance, A Large Matrix of Antineoplastic Agent Combinations from the National Cancer Institute (NCI-ALMANAC) contains 5232 drug pairs tested against 60 cancer cell lines^5^. Another resource provides drug responses measured for a panel of 39 cancer cell lines and 22 experimental drugs in all possible pairwise combinations and in combination with 16 approved drugs, totaling 583 compound pairs^6^. Other datasets are focused on a specific cell line, for example, 1833 bioactive drugs at 5 μm were tested in combination with temozolomide at 400 μm against a human glioblastoma cell line T98G^N^^7^. Furthermore, 1327 drug combinations from the CeMM library of unique drugs (CLOUD) dataset containing 308 prodrugs and active drugs^8^ were found effective against a human chronic myeloid leukemia cell line KBM-7^9^.

Meta-datasets collect and standardize the results of individual drug combination screening studies in order to enable a more efficient utilization of these data resources. For instance, DrugComb is an open-access data portal to 739,964 combinations of 8397 drugs tested on 2320 cell lines from 33 tissues^10,11^. It quantifies the degree of drug-drug interactions over the full dose–response matrix with several synergy scores, Bliss independence (BLISS), Highest single agent (HSA), Loewe additivity (LOEWE), and Zero interaction potency (ZIP)^12–14^. SYNERGxDB is a comprehensive dataset compiled from nine individual datasets containing 22,507 pairwise combinations of 1977 drugs tested on 151 cell lines from 15 tissues^15^. Similar to DrugComb, SYNERGxDB also provides standardized synergy scores, BLISS and ZIP. Finally, Dialog for Reverse Engineering Assessments and Methods (DREAM) Challenges partnered with AstraZeneca and the Sanger Institute to compile a dataset of 20,483 synergy scores for 910 drug combinations involving 118 anticancer drugs tested against 85 cancer cell lines^16^. This dataset also provides a quality assessment score for each combination, ranging from − 3 to 1, where 1 indicates a synergy between drugs in the combination. Along with the synergy data for drug combinations, the AZ-DREAM Challenges data comprise various molecular data, such as mutations, copy number variation, gene expression, and the tissue of origin. These datasets offer unparalleled opportunities to develop highly accurate ML models to predict drug synergistic effects.

Since the performance of supervised ML strongly depends on the quality, quantity, and the contextual subject of training data, the data scarcity problem is one of the most common challenges to develop robust ML models. To overcome this difficulty, data augmentation techniques are widely employed to expand the volume of available data. For instance, classical augmentation methods, such as image flipping, image rotation, noise injection, kernel filters, random erasing, and image mixing, are frequently used in the medical image analysis domain^17–22^. Data augmentation techniques gaining attention in the medical time series analysis domain^23^ include the time domain augmentation^24^, the time–frequency domain augmentation^25^, decomposition-based methods^26,27^, statistical generative models^28,29^, and learning-based methods^30–33^. In addition, more advanced deep learning-based augmentation techniques, including the feature space augmentation^34,35^, generative adversarial networks (GAN)-based augmentation^36–40^, the neural style transfer^41,42^, and meta-learning schemes^43–45^, have been proposed.

To combat overfitting in a neural network architecture with 60 million parameters for image recognition, two types of data augmentation were employed, label-preserving transformations and altering the intensities of the RGB channels in training images using Principal Component Analysis^46^. Indeed, these data augmentation techniques significantly reduced overfitting and improved performance, leading to the reduction in the top-1 error rate by more than 1%. CutMix is an interesting augmentation technique that combines regions from different images to create augmented samples^47^. CutMix improves model generalization by encouraging localization, providing diverse training examples, and enhancing model robustness against input corruption, as well as out-of-distribution detection performances. Augmenting training data with bilingual lexicon information was demonstrated to improve the performance of machine translation models on low-resource and unsupervised languages^48^. Three main types of lexical augmentation employed are codeswitching, lexical prompting, and raw token-pair training. Extensive experimentation results show that applying any of these augmentations to monolingual data yields substantial improvements, and that they can be combined for even greater effect.

Although image, language, and sequential data augmentation methods are well established, these approaches are, in principle, unsuitable to generate the heterogeneous data of cellular and molecular features for drug synergy prediction with supervised ML. On that account, a variety of domain-specific techniques have been developed. For instance, the fact that multiple simplified molecular-input line-entry system (SMILES) strings represent the same molecule was used to augment a molecular dataset of chemical species^49^ using the SMILES enumeration^50^. Further, data augmentation utilizing multiple SMILES representations for a single compound was demonstrated to enhance the prediction accuracy of various molecular properties, such as solubility, lipophilicity, and bioactivity, irrespective of the specific machine learning model employed or the size of the dataset^51^. Another study doubled the size of a training dataset to predict anticancer drug synergism based on NCI-ALMANAC by generating duplicates with the reverse order of drugs^52^. Data up-sampling was also applied to increase the number of minor class instances for phenotype-based virtual screening of anticancer drug combinations^53^. Finally, an example of a deep learning-based data augmentation technique is the uniform graph convolutional network (UGCN)^54^. It employs a drug representation based on atomic interactions within organic compounds rather than hand-crafted features, such as molecular fingerprints, and string-based features, such as SMILES. UGCN can be used to augment chemical data by randomly sampling multiple complementary graphs for a single drug.

Despite the encouraging results reported for the abovementioned data augmentation techniques for drug synergy prediction, many of existing methods either are too general (up-sampling) or consider only drug structural information (SMILES enumeration and UGCN). To address these issues, we devised a new augmentation approach combining the drug chemical similarity with the system-level information on drug-target interactions. This approach employs a novel similarity metric, the drug action/chemical similarity (DACS) score, taking into account not only the chemical characteristics of drugs, but also their molecular targets. Applying the DACS score to augment the AZ-DREAM Challenges data with new compounds from PubChem^55^ significantly increased the size and diversity of the training dataset for drug synergy prediction. To the best of our knowledge, this methodology represents the first systematic and effective protocol to augment a synergy dataset simultaneously utilizing the information on drug chemical structures and their protein targets. As a proof of concept, the augmented dataset was used to train several ML models demonstrating a higher accuracy of drug synergy prediction compared to those models trained on the original AZ-DREAM Challenges data.

## Results

### Similarity measure for cellular responses to drug treatment

**Figure 1:**
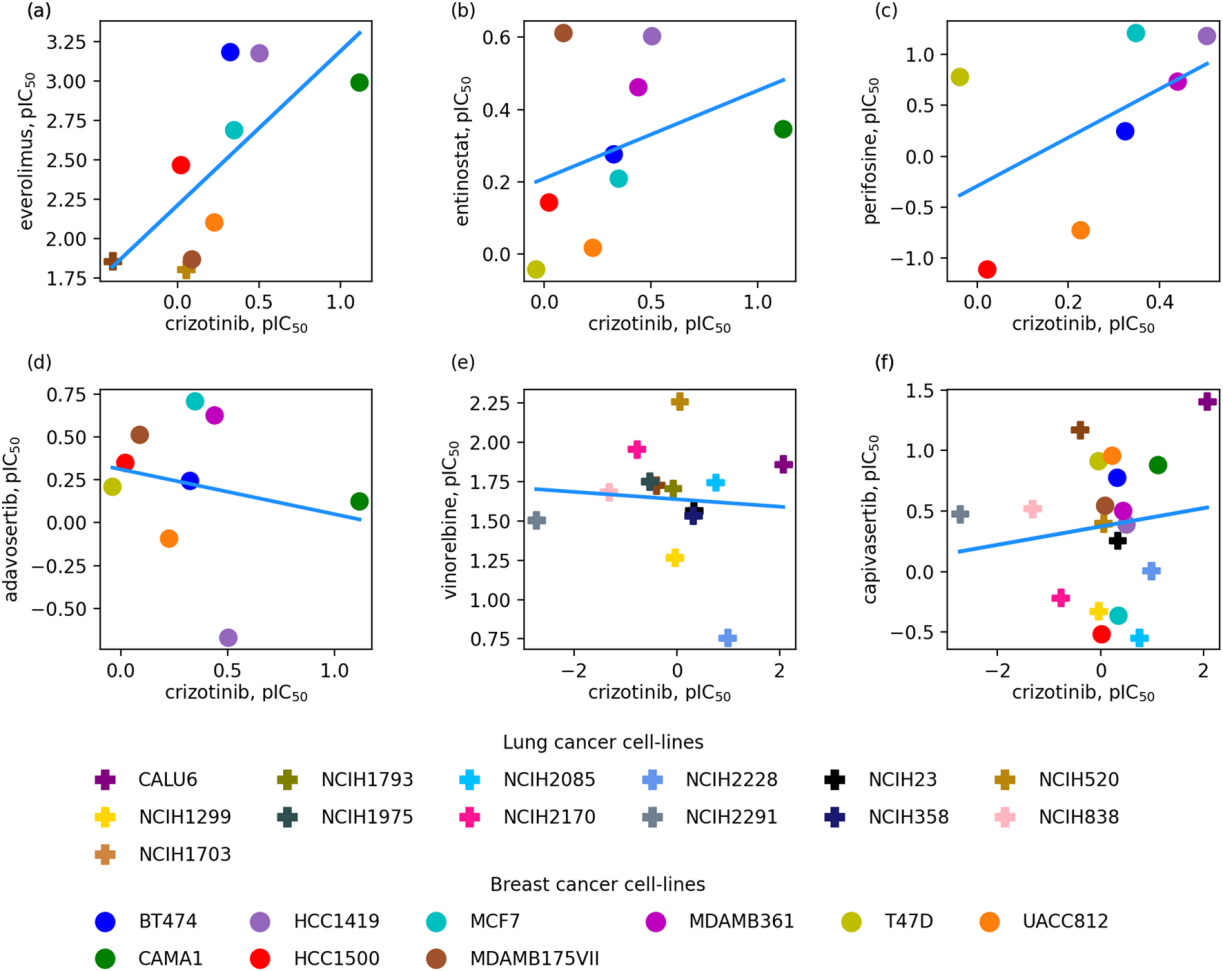
Similarity of pharmacological effects of two drugs quantified by the Kendall τ correlation coefficient. pIC_50_ values for the monotherapy treatments of multiple cancer cell lines with crizotinib are plotted against those for (**A**) everolimus, (**B**) entinostat, (**C**) perifosine, (**D**) adavosertib, (**E**) vinorelbine, and (**F**) capivasertib. (**A**, **B**, and **C)** are examples of the positive correlation, whereas (**D**, **E**, and **F)** represent the negative correlation. Individual breast cancer cell lines are shown as solid circles and lung cancer cell lines as solid plus signs.

During the data augmentation, new drug combinations are generated by replacing drugs with those molecules triggering similar pharmacological responses. The similarity of pharmacological effects of two drugs is quantified by the Kendall τ correlation coefficient between pIC_50_ values for the monotherapy treatments of multiple cancer cell lines. A positive value of Kendall τ indicates that two drugs have similar pharmacological effects in terms of the inhibition of the cancer growth, whereas a negative correlation and the lack of correlation point to different cellular responses to drug treatment. This concept is illustrated in Fig. 1 for crizotinib, a tyrosine kinase inhibitor used for the treatment of non-small cell lung carcinoma (NSCLC)^56^, paired with six other anti-cancer drugs. Figures 1A–C are examples of a positive correlation between crizotinib and everolimus (Kendall τ of 0.50), entinostat (Kendall τ of 0.44), and perifosine (Kendall τ of 0.42), respectively. Everolimus, a derivative of sirolimus with cell proliferation and immunosuppressive properties, is used in combination with other anticancer agents for the treatment of kidney and breast cancer, and neuroendocrine tumors of gastrointestinal and lung origins^57^. Entinostat, a benzamide derivative with the antineoplastic activity, and perifosine, an allosteric AKT inhibitor with the antiglycolytic activity, are used for the treatment of NSCLC^58,59^. According to the analysis of pIC_50_ values against multiple cancer cell lines, these three drugs have similar profiles to that of crizotinib, i.e., they inhibit the growth of the same cancer cell lines and are ineffective against the same group of cell lines as well.

In contrast, cellular responses of crizotinib are uncorrelated with that of adavosertib (Fig. 1D, Kendall τ of − 0.06), vinorelbine (Fig. 1E, Kendall τ of − 0.03), and capivasertib (Fig. 1F, Kendall τ of − 0.01). Adavosertib is a tyrosine kinase WEE1 inhibitor used to improve the outcome in triple-negative breast cancer^60^, vinorelbine is an agent to treat NSCLC and breast cancer^61^, and capivasertib is AKT inhibitor used in the treatment of breast cancer^62^. Since these drugs have uncorrelated pharmacological effects, they cannot be used to replace crizotinib during the data augmentation process. The analysis of cellular responses with the Kendall τ is versatile and can be applied when two drugs have been tested on at least two common cell lines, otherwise the value of the Kendall τ is set to 0. The similarities of pharmacological effects between crizotinib and everolimus, entinostat, perifosine, adavosertib, vinorelbine, and capivasertib were calculated based on 7 + 2, 9 + 0, 7 + 0, 9 + 0, 0 + 13, and 9 + 10 common (breast + lung) cell lines, respectively.

### Relation between drug similarity and pharmacological effects

**Figure 2:**
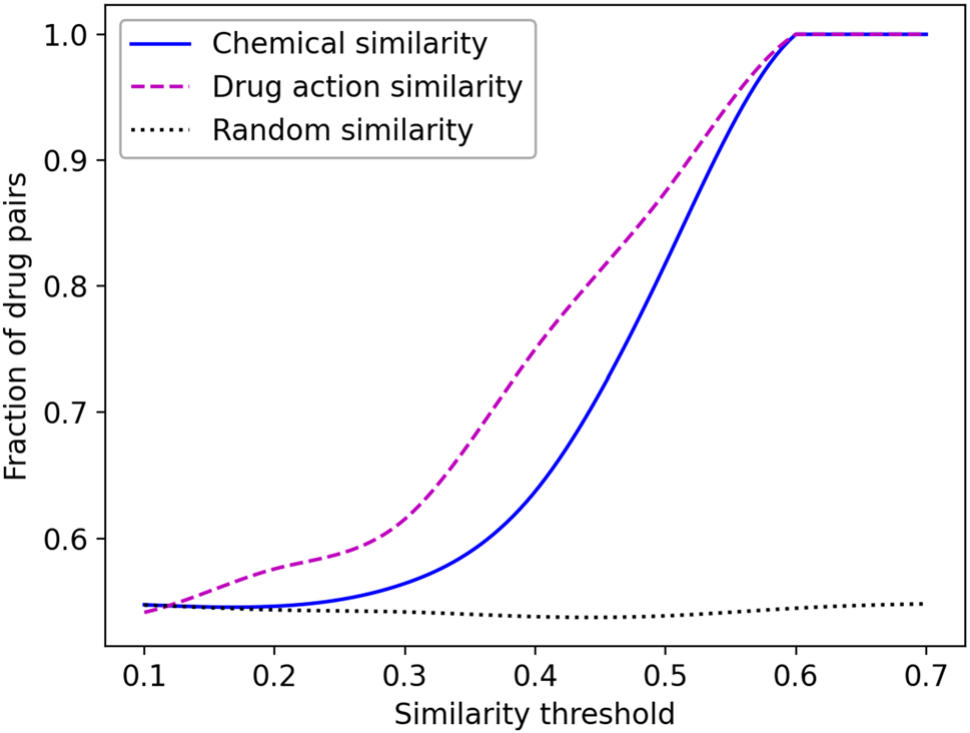
Fraction of drug pairs with positively correlated pharmacological effects as a function of their similarities. The chemical similarity (solid blue line) is measured with the Tanimoto coefficient between drug FP2 fingerprints. The drug action similarity (dashed purple line) is quantified with the Matthews correlation coefficient between target proteins in the IHP-PING protein–protein interaction network. Random similarity (dotted black line) is obtained by assigning a random number between 0 and 1.

Next, we investigate how similar two drugs need to be in order to trigger similar pharmacological effects. This analysis is performed for 4753 $\left(98{\text{C}}2\right)$ possible pairs of 98 drugs in the AZ-DREAM Challenges dataset. Pharmacological responses are quantified with the Kendall τ correlation coefficient, whereas the drug similarity is measured with two metrics. The first score is the drug chemical similarity calculated as the Tanimoto coefficient (TC) between FP2 fingerprints^63^. Figure 2 (solid blue line) shows that, as expected, the fraction of drug pairs with the positive Kendall τ increases with the increasing chemical similarity and reaches a value of 1.0 for the TC threshold of 0.6. The second metric is the drug action similarity computed as the Matthews correlation coefficient (MCC)^64^ between target proteins in the protein–protein interaction (PPI) network from the IHP-PING dataset^65^. Similar to the TC, the fraction of drug pairs with the positive Kendall τ also increases with the increasing MCC reaching 1.0 for the MCC threshold of 0.6 (Fig. 2, dashed purple line). For comparison, increasing the threshold for a random similarity does not increase the fraction of drug pairs with the positive Kendall τ (Fig. 2, dotted black line).

### Drug action/chemical similarity score

**Figure 3:**
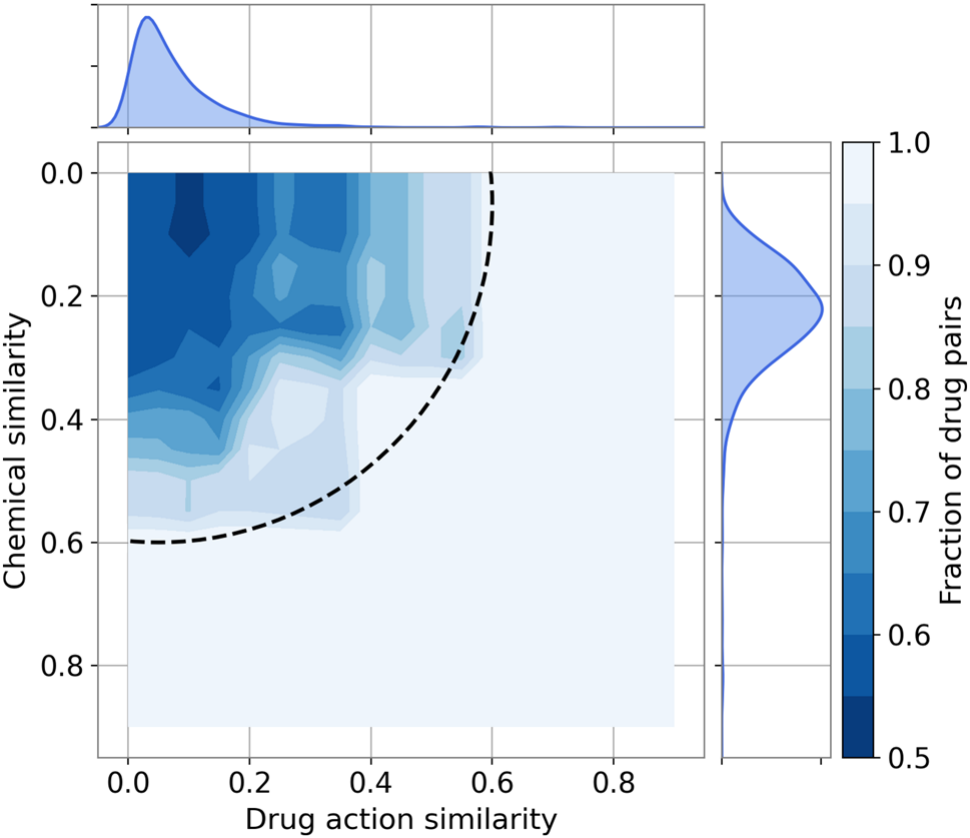
Heatmap of the fraction of drug pairs with positively correlated pharmacological effects. The fraction of drug pairs with the positive Kendall τ is displayed according to the color scale on the right. One-dimensional histograms show the distributions of the chemical similarity (a subplot on the right) and the drug action similarity (a subplot on the top). The dashed quarter circle represents a DACS threshold of 0.6.

Analyses presented above demonstrate that both chemical and drug action similarities can be used for data augmentation. However, their combination could potentially cover a larger chemical space than individual similarities while ensuring that the pharmacological profiles of drugs selected for augmentation are highly similar to those of their parent molecules. Therefore, we combined TC and MCC into a new metric, the drug action/chemical similarity (DACS) score. Figure 3 shows the relation between the DACS score and the fraction of drug pairs with the positive Kendall τ as the spatial heatmap in two dimensions corresponding to the individual similarities. The dark blue section in the upper left corner of the heatmap corresponds to the area of a low positive correlation, whereas the light blue section shows the combination of individual similarities resulting in a high positive correlation. The DACS score can be represented as a quarter circle in Fig. 3 (dashed black line). For example, above a DACS threshold of 0.6, as many as 85.7% drug pairs have a positive Kendall τ correlation.

### Dataset augmentation with DACS

**Figure 4:**
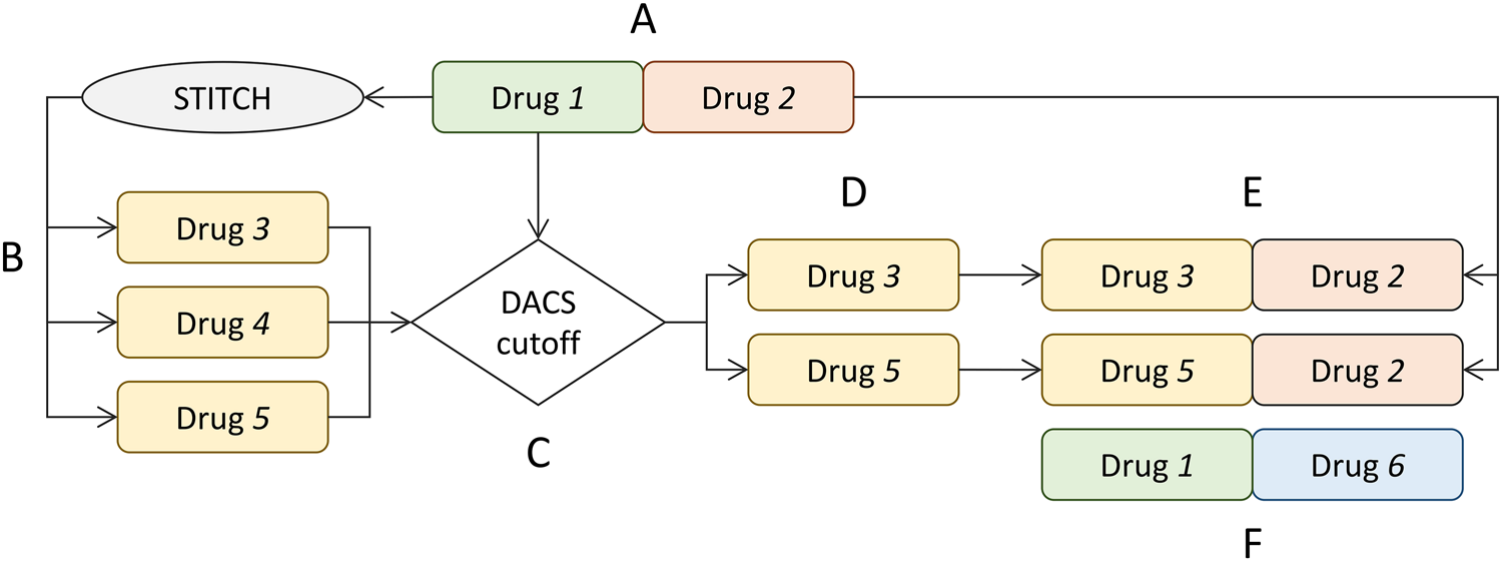
Flowchart of the augmentation procedure. The procedure starts with an original drug pair *1*:*2*, in which drug *1*, represented by a green rounded box, is to be replaced first (**A**). Candidate molecules *3*, *4*, and *5*, represented by yellow boxes, are selected from the STITCH database (**B**). DACS scores for compounds *3*, *4*, and *5* are calculated against drug *1* (**C**) and those molecules with scores larger than a cutoff are retained (**D**). These compounds are then combined with drug *2* creating augmented instances *3*:*2* and *5*:*2* (**E**). The same procedure is then applied to replace drug *2*, represented by an orange box. This generates more augmented instances containing drug *1*, such as an augmented pair *1*:*6*, in which molecule *6*, represented by a cyan box, is a substitute for drug *2* (**F**). The class of augmented instances (either synergistic or antagonistic) is transferred from the original drug pair *1*:*2*.

The DACS metric is used as a guide to find the optimal number of new instances to be generated for the synergy dataset according to a procedure presented in Fig. 4. Each instance in the AZ-DREAM Challenges dataset consists of a pair of drugs targeting a cell line with a particular synergy score (Fig. 4A, drug pair *1*:*2*). During the augmentation procedure, candidate molecules to replace one drug in a pair are identified in the STITCH database^66^ (Fig. 4B, drugs *3*, *4*, and *5*). Next, DACS scores against the drug to be replaced are calculated (Fig. 4C) and those molecules having scores larger than a cutoff are selected (Fig. 4D, drugs *3* and *5*). The original drug is then replaced by the selected molecules to create augmented pairs (Fig. 4E, drug pairs *3*:*2* and *5*:*2*). This procedure is repeated for the second drug in the original pair creating more augmented instances (Fig. 4F, drug pairs *1*:*6*).

**Figure 5:**
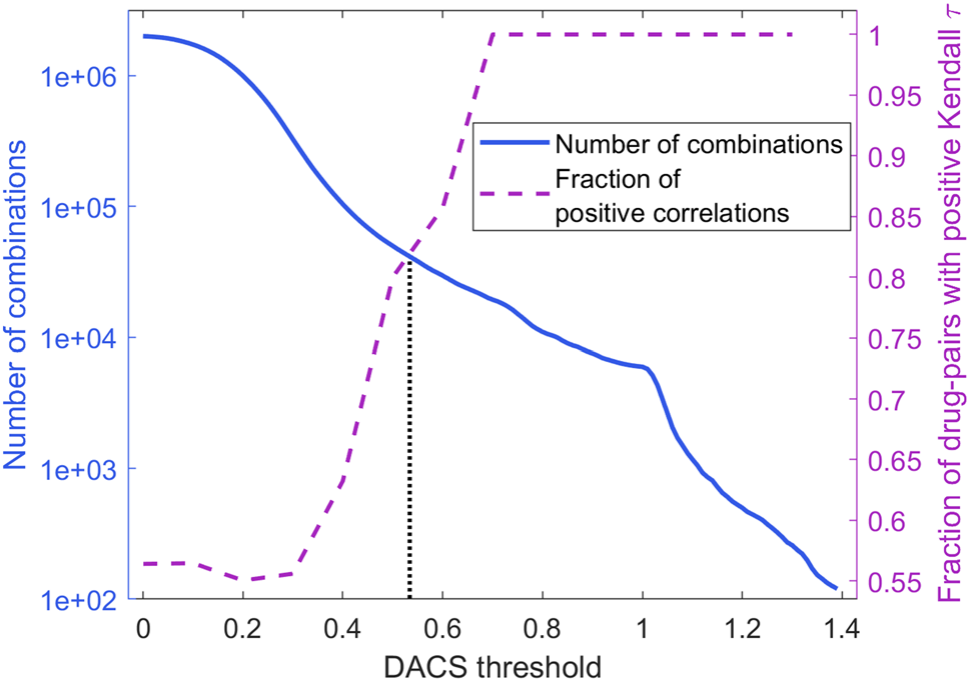
Selection of the optimal DACS threshold for data augmentation. The solid blue curve represents the number of potential substitutes for the original 98 drugs that can be found in the STITCH database as the DACS threshold is increased. The dashed purple line represents the change in the fraction of drug pairs with the positive Kendall τ as the DACS threshold is increased. The vertical dotted line marks the DACS threshold optimizing these two quantities.

The selection of a cutoff for DACS scores between the original drug to be replaced and the candidate substitute compounds is critical to create high-quality augmented instances. On that account, we conducted an analysis of the fraction of new drugs having similar pharmacological profiles to their parent molecules and the number of new instances that can be obtained from the STITCH database at different DACS similarity thresholds. Figure 5 shows that these two quantities are inversely related, i.e., increasing the DACS similarity threshold results in a higher chance of substitute compounds to trigger similar pharmacological responses (dashed purple line), however, at the same time, fewer molecules can be used to augment the dataset (solid blue line). The intersection point marked by a dotted black line in Fig. 5 represents the DACS cutoff of 0.53, at which the majority of substitute drugs (82%) have similar pharmacological profiles to their parent molecules and as many as 42,225 new drugs can be obtained from the STITCH database to augment the synergy dataset. Applying this threshold to replace one molecule in a drug pair in the AZ-DREAM Challenges dataset of 8798 instances produces an augmented dataset of 6,016,697 drug pairs annotated with synergy scores against various cancer cell lines.

**Figure 6:**
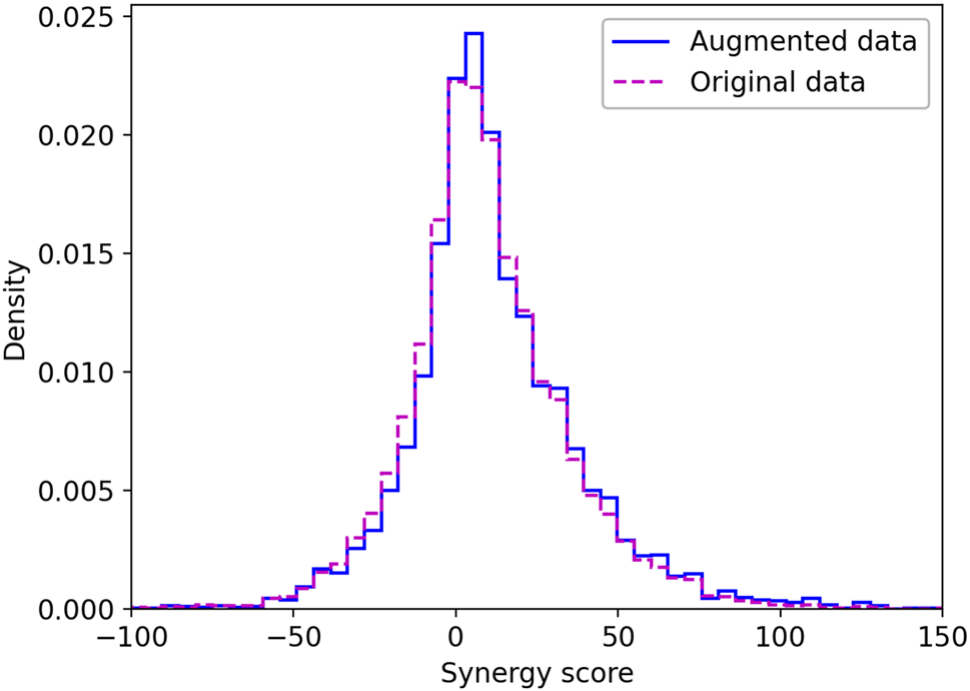
Distribution of synergy score across drug synergy datasets. The step histogram in purple dashed line shows the distribution of synergy scores in the original AZ-DREAM Challenges data, whereas the step histogram in blue solid line shows the distribution of synergy scores in the augmented dataset.

Ideally, the distribution of synergy values across the augmented dataset should be the same as for the AZ-DREAM Challenges dataset. Figure 6 shows that these two distributions indeed are similar; the average synergy score ± standard deviation is 9.9 ± 26.1 for the AZ-DREAM Challenges dataset and 12.1 ± 28.5 for the augmented dataset. In addition, we compare various physicochemical properties of drugs present in the original and augmented dataset to those calculated for a set of 27,385 molecules selected randomly from the STITCH database^66^. Indeed, the original and augmented drugs have similar octanol–water partition coefficient (log*P*, 3.6 ± 2.0 and 3.8 ± 1.8), the number of hydrogen bond donors (HBD, 2.0 ± 1.2 and 2.0 ± 1.6) and acceptors (HBA, 6.8 ± 2.6 and 5.8 ± 2.4), and the Quantitative Estimate of Druglikeness^67^ (QED, 0.48 ± 0.18 and 0.49 ± 0.20). For comparison, log*P*, HBD, HBA, and QED for random molecules are 3.2 ± 2.4, 1.9 ± 1.9, 5.0 ± 2.7, and 0.50 ± 0.22, respectively. These analyses demonstrate that the augmented dataset does not contain artifacts, such as molecules with certain physicochemical properties, that could potentially bias the training of machine learning models toward a particular effect (either synergism or antagonism).

### Drug synergy prediction with machine learning

Finally, we investigate whether training machine learning against the augmented data achieves a better classification performance than training against the original AZ-DREAM Challenges dataset. Four state-of-the-art machine learning methods are employed, Logistic Regression (LR)^68,69^, Support Vector Machines (SVM)^70,71^, Random Forest (RF)^72^, and Gradient Boosting Trees (GBT)^73^. Following the original publication^16^, drug pairs having synergy scores higher than 20 are labelled synergistic and those having synergy scores lower than − 20 are labelled antagonistic. First, we performed a fivefold cross-validation by randomly splitting the dataset into 5 subsets. Note that the augmented data are only used to train machine learning models, which are then validated against AZ-DREAM Challenges instances. Table 1 shows the classification performance evaluated with several metrics. Encouragingly, the performance of classifiers is improved when models are trained against the augmented data and the random-split validation is employed. For instance, the area under the receiver operating characteristic plot (AUC) increased from 0.802 to 0.809 for RF and from 0.859 to 0.863 for GBT classifiers.

**Table 1:** Performance of machine learning in the prediction of drug synergistic effects.

Classifier	Validation protocol	Dataset	ACC	TPR	FPR	PPV	AUC	MCC	F1-score
LR	Random-split	Original	0.752	0.768	0.302	0.893	0.809	0.417	0.826
		Augmented	0.756	0.770	0.292	0.897	0.811	0.427	0.829
	Tissue-based	Original	0.631	0.718	0.509	0.769	0.659	0.200	0.728
		Augmented	0.637	0.711	0.475	0.777	0.661	0.221	0.729
SVM	Random-split	Original	0.745	0.755	0.287	0.897	0.803	0.414	0.819
		Augmented	0.750	0.757	0.271	0.902	0.803	0.429	0.823
	Tissue-based	Original	0.619	0.736	0.546	0.761	0.671	0.191	0.721
		Augmented	0.651	0.771	0.560	0.765	0.674	0.208	0.751
RF	Random-split	Original	0.754	0.787	0.358	0.879	0.802	0.392	0.831
		Augmented	0.757	0.788	0.347	0.882	0.809	0.402	0.832
	Tissue-based	Original	0.667	0.811	0.644	0.749	0.647	0.173	0.769
		Augmented	0.705	0.866	0.659	0.758	0.685	0.226	0.801
GBT	Random-split	Original	0.833	0.921	0.457	0.869	0.859	0.503	0.894
		Augmented	0.840	0.927	0.445	0.873	0.863	0.524	0.899
	Tissue-based	Original	0.716	0.930	0.803	0.734	0.688	0.176	0.815
		Augmented	0.736	0.940	0.743	0.750	0.734	0.260	0.828

*ACC* accuracy, *TPR* recall, *FPR* false positive rate, *PPV* precision, *AUC* area under the receiver operating characteristic plot, *MCC* Matthews correlation coefficient.

Two protocols are employed utilizing the random-split of the data and the tissue-based cross-validation. The performance of Logistic Regression (LR), Support Vector Machine (SVM), Random Forest (RF), and Gradient Boosting Trees (GBT) classifiers is evaluated against the original AZ-DREAM Challenges data and the augmented dataset.

Although a random-split cross-validation is often used to assess the performance of drug synergy predictors ^16^ , it leads to a significant overlap between training and validation subsets because those instances involving similar cell lines are present in both sets. Consequently, the trained model is going to have only a weak ability to generalize to unseen data, even though the validation accuracy may seem high. In order to mitigate this issue and more reliably evaluate the performance of machine learning trained on drug synergy data, we conducted a tissue-based cross-validation in which each fold comprises a particular tissue (or a group of tissues). This protocol has been shown to eliminate the overlap between training and validation subsets allowing for an unbiased assessment of the capabilities of machine learning to extract the information from input data ^74^.

**Figure 7:**
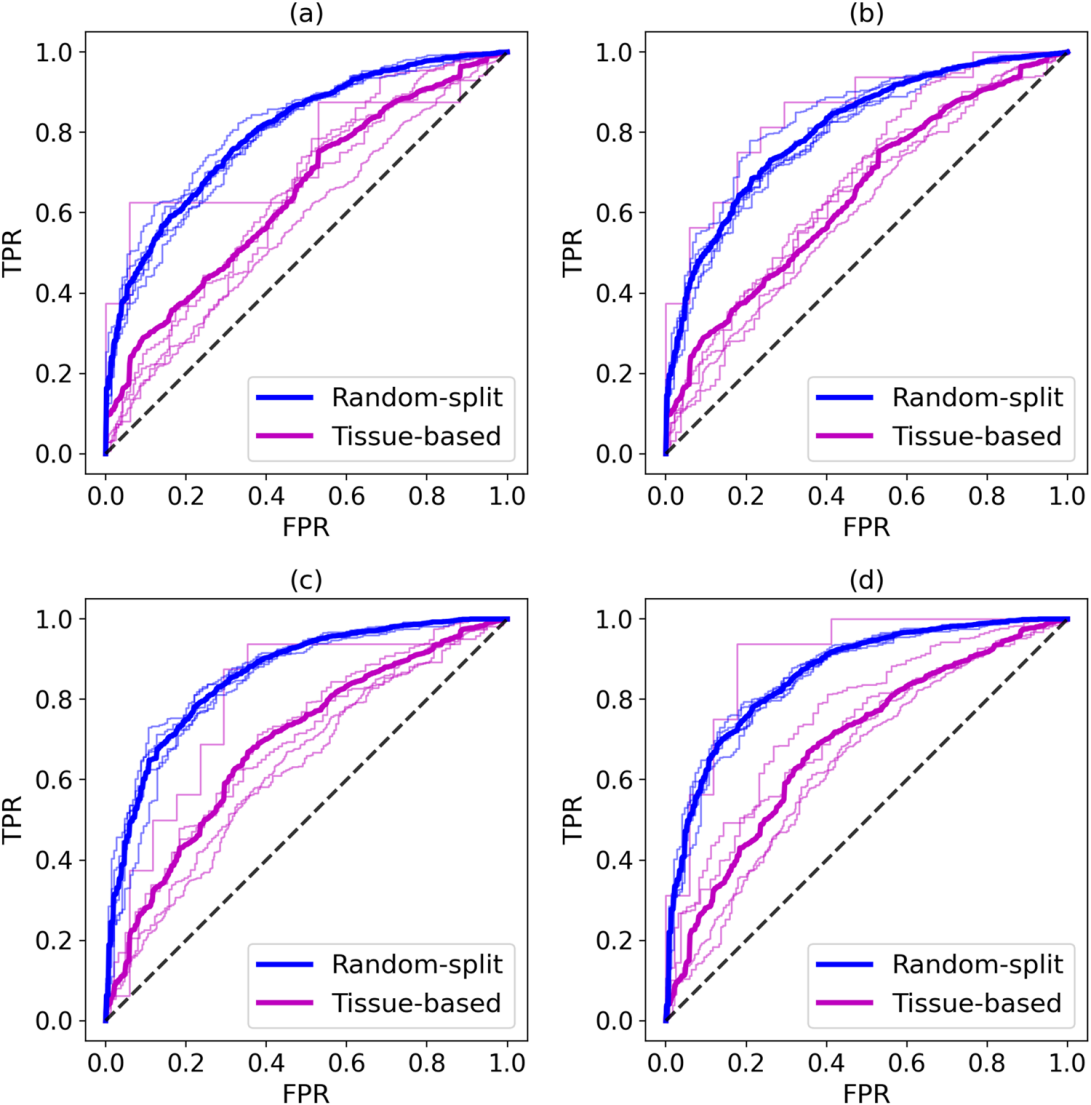
Performance of machine learning in the prediction of drug synergistic effects. Receiver operating characteristics plots for the Random Forest classifier against (**A**) the original AZ-DREAM Challenges data and (**B**) the augmented dataset, and for the Gradient Boosting Trees classifier against (**C**) the original AZ-DREAM Challenges data and (**D**) the augmented dataset. Blue lines were calculated for the random-split protocol, while purple lines were obtained for the tissue-based cross-validation. Thick lines show the mean performance averaged over individual folds represented by thin lines.

Table 1 and receiver operating characteristic plots presented in Fig. 7 show that applying the more rigorous tissue-based validation protocol decreases the performance of machine learning predicting drug synergistic effects. However, this evaluation is more reliable because it better mimics a real scenario in which machine learning is applied to predict drug synergistic effects for unseen data, i.e., drug combinations against cell lines originating from tissues that have not been used to train the classifier. With this cross-validation protocol, machine learning trained on the augmented data yields even higher improvements in terms of the classification accuracy compared to models trained on the original AZ-DREAM Challenges dataset. For example, the AUC increased from 0.647 to 0.685 for RF and from 0.688 to 0.734 for GBT classifiers.

Table 2 shows AUC scores for each tissue fold and tree-based models trained on both the original and the augmented datasets. The comparison of AUC scores reveals that incorporating the augmented data into the training process systematically improves the classification performance regardless of the tissue type. In general, these findings indicate that incorporating augmented data can provide enhanced information for training machine learning models in a more effective manner.

**Table 2:** Area under the receiver operating characteristic plot (AUC) scores for each fold in the tissue-based cross-validation.

Classifier	Dataset	Breast tissue	Digestive system	Excretory system	Respiratory system	Other
RF	Original	0.574	0.628	0.650	0.636	0.746
	Augmented	0.613	0.640	0.664	0.658	0.849
GBT	Original	0.637	0.675	0.631	0.704	0.794
	Augmented	0.648	0.715	0.649	0.752	0.904

The performance of Random Forest (RF) and Gradient Boosting Trees (GBT) classifiers is reported for the original and the augmented AZ-DREAM Challenges datasets.

### Classification of instances with ambiguous synergy scores

**Figure 8:**
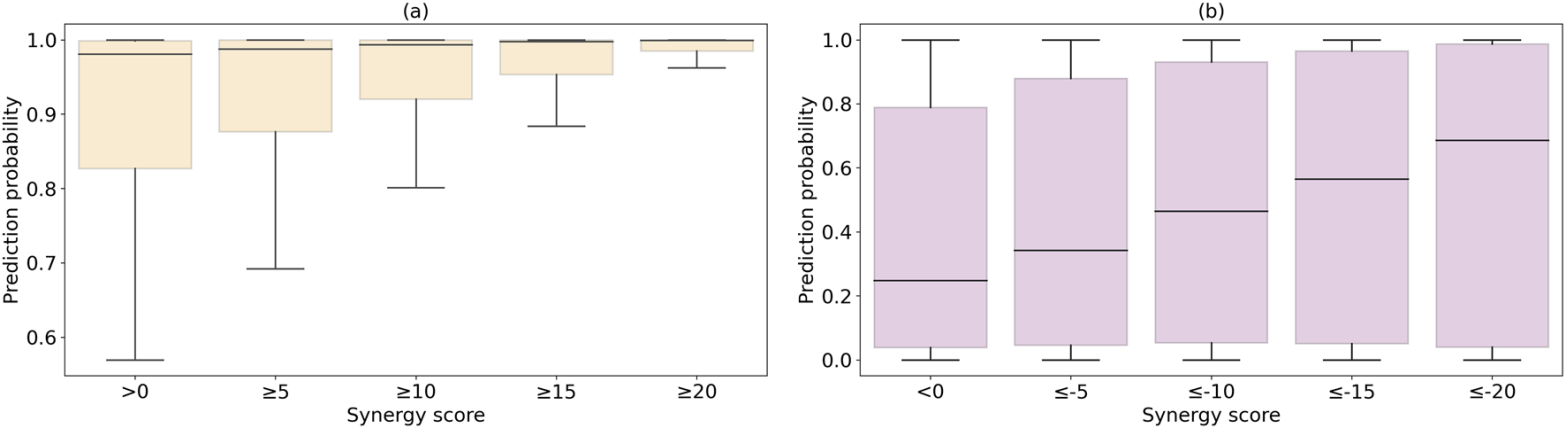
Distribution of prediction probabilities across varying degrees of drug synergy. Predictions are generated by the Gradient Boosting Trees classifier for (**A**, light yellow) positive instances with an increasing threshold for the synergy scores from $>0$ to $\ge 20$ and (**B**, light purple) negative instances with a decreasing threshold for the synergy scores from $<0$ to $\le -20$. Boxes end at quartiles *Q*_1_ and *Q*_3_, *Q*_2_ is the median. Whiskers extend from *Q*_1_ and *Q*_3_ to the most extreme data points within ${Q}_{1}-1.5\times IQR$ and ${Q}_{3}+1.5\times IQR$, respectively, where *IQR* is the inter-quartile range.

The robustness of ML models stems from the foundation laid by the quality of the training data, ensuring that they can effectively handle diverse and complex scenarios with a high degree of accuracy. When a machine learning model encounters instances with ambiguous labels, it adapts by making predictions that are less confident for such cases. To illustrate this phenomenon, we evaluate the capability of the trained GBT model to handle instances with unclear class labels by assessing its performance across a spectrum of synergy scores. The GBT model was selected because its performance in fivefold cross-validation against instances with reliable synergy scores $\ge 20$ (synergistic cases) and $\le -20$ (antagonistic cases) is better than those of LR, SVM, and RF. Figure 8 shows the distribution of prediction probabilities reported by the GBT model for drug combinations selected from the AZ-DREAM Challenges dataset with a varying degree of synergy scores with the corresponding statistics reported in Table 3.

**Table 3:** Statistics for the distribution of prediction probabilities across varying degrees of drug synergy.

Quartile	Synergy score (positives)					Synergy score (negatives)				
	$>0$	$\ge 5$	$\ge 10$	$\ge 15$	$\ge 20$	$<0$	$\le -5$	$\le -10$	$\le -15$	$\le -20$
*Q*_1_	0.827	0.877	0.921	0.954	0.985	0.039	0.046	0.053	0.050	0.041
*Q*_2_	0.981	0.987	0.994	0.998	0.999	0.248	0.342	0.466	0.564	0.687
*Q*_3_	0.999	0.999	1.000	1.000	1.000	0.789	0.879	0.931	0.965	0.989

Predictions are generated by the Gradient Boosting Trees classifier for positive instances with an increasing threshold for the synergy scores from $>0$ to $\ge 20$ and negative instances with a decreasing threshold for the synergy scores from $<0$ to $\le -20$. *Q*_2_ is the median.

Including ambiguous labels represented by synergy scores close to 0 lowers the confidence, and the model attempts to reflect this uncertainty in its predictions. For instance, Fig. 8A shows that the median (*Q*_2_) prediction probability is 0.981 when the most ambiguous positive cases with the synergy score of $>0$ are included, while it is as high as 0.999 when the model is applied to only the most reliable positive cases with the synergy score of $\ge 20$. This trend can also be observed for negative instances (Fig. 8B), for which the median prediction probability increases from 0.248 for the most ambiguous cases with the synergy score of $<0$ to 0.687 for the most reliable cases with the synergy score of $\le -20$. Another indication of the lack of strong prediction confidence when instances having unclear labels are included is the increased spread of prediction probabilities. Indeed, wider interquartile ranges (*Q*_3_-*Q*_1_) are observed when ambiguous positive cases are considered compared to those obtained for the most reliable drug combinations only. For negative cases, *Q*_2_ and *Q*_3_ values decrease as more unclear instances are included, meaning there is a concentration of prediction probability towards the lower values, which signifies the declined prediction confidence for those instances and a diminished level of assurance in the ability to assign accurate classifications by the model.

### Evaluation against “unseen” data

To further evaluate the generalizability of a model trained on the AZ-DREAM Challenges augmented data, we conducted the performance evaluation against an independent dataset of 250 drug combinations selected from DrugCombDB^75^. It is important to note that since drugs in this set are chemically dissimilar to those in the AZ-DREAM Challenges dataset, DrugCombDB instances can be regarded as “unseen” data. In this analysis, two GBT models were trained, one using the original AZ-DREAM Challenges data and the other using both the original and augmented instances. A GBT model trained solely on the original data correctly classified only 76/250 drug combinations (12 synergistic and 64 antagonistic) yielding the accuracy of 0.30 and a high false positive rate (FPR) of 0.73. In contrast, a GBT model that incorporated augmented data during training correctly predicted 141/250 drug combinations (11 synergistic and 130 antagonistic) achieving a much higher accuracy of 0.56 and a significantly lower FPR of 0.45. This improved performance by employing augmented instances highlights the importance of data augmentation techniques in enhancing the ability of machine learning models to generalize to new drug synergy data. Through exposure to a comprehensive and diverse dataset, the model acquired improved pattern recognition capabilities and achieved more accurate classifications, resulting in an enhanced reliability for drug synergy predictions in a real-world application scenario.

## Discussion

In this study, we devised a data augmentation protocol to solve the data scarcity problem in predicting synergistic effects of anti-cancer drug combinations with machine learning models. The augmentation protocol expands the synergy dataset by replacing a compound in a drug combination instance with another molecule having highly similar pharmacological effects. This is achieved through the use of the DACS similarity metric between two drugs, which incorporates both chemical structure and drug action similarities. Compared to existing techniques used in synergy data augmentation, such as the upsampling^53^, the SMILES enumeration^50^, and the reverse order of drugs^52^, which essentially duplicate the existing data points, our approach expands the dataset by including new, unbiased instances. As a results, this augmentation methodology not only enriches the available data points, but also enhances the diversity of the data, which is highly beneficial to improve the generalizability of machine learning models. Additionally, in contrast to other augmentation approaches involving a learning process^54^, our method generates data points in a shorter amount of time.

While random-split cross-validation is frequently utilized for data partitioning, it may lead to tissue-level overlap and elevate the possibility of model overfitting, particularly when dealing with data containing multiple cell lines from the same tissue. The reason for this is that those instances involving similar cell lines tend to have comparable feature representations, such as gene expression profiles and the gene-disease association. The overlap is likely going to occur when these instances are present in both the training and validation sets^76^. In such cases, the trained model may exhibit a strong performance due to the presence of overlapping data, but it will not perform well on novel, unseen data. Consequently, the model may be overestimated in terms of its true performance and fail to generalize to other datasets. On the other hand, a tissue-based cross-validation can effectively eliminate the data overlap issue. By excluding all instances originating from a validation tissue from the training set for each fold, the generalizability of a machine learning model can be properly evaluated.

Tree-based models (RF and GBT) employed in this study are robust, interpretable, and widely adopted by AZ-DREAM Challenges participants^16^. These models have the ability to deal with complex non-linear input–output relationships and can handle sizable datasets to a certain degree. Neither tree-based models nor other classifiers like LR and SVM are designed to exploit intricate relationships between features. This limitation is especially notable when dealing with heterogeneous features, including protein–protein interactions, gene expression levels, and drug-protein associations. In such cases, these models may struggle to find the optimal decision boundaries, generally leading to an unsatisfactory performance. Neural networks, on the other hand, are better equipped to handle diverse data types and can learn complex relationships between features with hidden layers and non-linear activation functions. This ability to integrate multiple heterogeneous data into a single model can often result in an improved performance compared to tree-based models. Our future research will concentrate on exploring this aspect.

The augmentation protocol devised in this study is not limited to anti-cancer drug data can be used to expand other synergy datasets as well; it has the potential to become a universal tactic for enhancing datasets in drug discovery and related fields. This could result in a greater amount of data being accessible and ultimately lead to better research results. Furthermore, the developed new drug similarity measure, the DACS score, improves the way drug similarity is assessed. By integrating both structural and target similarities, DACS provides a more exhaustive and inclusive perspective on drug similarity compared to traditional methods that only examine a single aspect, such as the chemical similarity. By offering a more holistic approach to analyzing and evaluating the similarities between drugs, DACS can help improve the accuracy and efficiency of the drug discovery process.

Deep learning, with its ability to dissect complex data and reveal underlying patterns and relationships, has become a pivotal tool in the field of pharmacology and drug development^77,78^. The varied and comprehensive synergy dataset created in this study has the potential to significantly aid deep learning models by offering a diverse range of data for training purposes. The utilization of sufficient data enables deep learning algorithms to recognize intricate relationships and connections among cellular, molecular, and biological system-level features, thereby elevating the precision and efficacy of synergistic effect predictions. Moreover, an extensive and varied dataset reduces the risk of overfitting, a common issue where models become too reliant on limited training data and struggle to generalize to new data. Thus, the utilization of a comprehensive synergy dataset can lead to more robust and dependable deep learning models and ultimately, more advanced outcomes in drug discovery and related fields.

In addition to being used in deep learning-based drug discovery, the proposed anti-cancer drug synergy dataset has the potential to facilitate other applications, such as drug repositioning, drug target identification, toxicity analysis, the modeling of drug interactions, systems pharmacology, and precision medicine. By providing valuable insights into the interactions between drugs, targets, and biological systems, the synergy data can contribute to the development of more effective and safer pharmaceutics. Overall, the wide-ranging possibilities arising from this study may have significant implications for the drug discovery and development field. Ultimately, this could result in the creation of novel therapeutic approaches for a range of diseases.

## Methods

### Similarity of drug pharmacological effects

The Kendall τ rank correlation coefficient is employed to measure the ordinal association between the pharmacological effects of two drugs against a set of cell lines. First, common cell lines targeted by both drugs are identified and two lists ranked by pIC_50_ values for monotherapy treatments are calculated. Next, the value of the Kendall τ accounting for ties $\left({\tau }_{b}\right)$^79,80^ is computed:

1 $${\tau }_{b}=\frac{{n}_{c}-{n}_{d}}{\sqrt{\left({n}_{c}+{n}_{d}+{n}_{1}\right)\left({n}_{c}+{n}_{d}+{n}_{2}\right)}}$$

where ${n}_{c}$ is the number of concordant cell line pairs (having the same order in both drug lists), ${n}_{d}$ is the number of discordant cell line pairs (having different order in both drug lists), ${n}_{1}$ is the number of pairs tied only in the first list, and ${n}_{2}$ is the number of pairs tied only in the second list. ${\tau }_{b}$ of $+1$ indicates a perfectly positive association, i.e., the two drugs having the same pharmacological effects in terms of the inhibition of the cancer growth across multiple common cell lines. A value of $-1$ indicates a perfectly negative association, i.e., the opposite pharmacological effects, and a value of $0$ indicates the lack of any association. The Kendall τ coefficient is calculated when pIC_50_ values are available for monotherapy treatments of at least two common cell lines, otherwise it is set to $0$.

### Similarity of drug molecular mechanism of action

Similarity of the mechanism of action of two drugs is quantified with the MCC^64^ computed for 19,968 proteins in the IHP-PING dataset^65^ according to chemical-protein associations obtained from the STITCH database^66^:

2 $$MCC= \frac{\left(T\times N\right)-\left(A\times B\right)}{\sqrt{\left(T+A\right)\left(T+B\right)\left(N+A\right)\left(N+B\right)}}$$

where $T$ is the number of proteins targeted by both drugs, $N$ is the number of proteins not targeted by any drug, $A$ is the number of proteins only targeted by the first drug, and $B$ is the number of proteins only targeted by the second drug. MCC ranges from $-1$ to $+1$ with high positive values indicating a significant overlap between the molecular targets of two drugs, thus a similar mechanism of action. The MCC for a pair of drugs having different mechanisms of action is going to be around $0$.

### Drug action/chemical similarity score

The DACS measure provides a convenient and informative way to combine the drug structure similarity with the similarity of the molecular mechanisms of action. It is calculated as:

3 $$DACS= \sqrt{{TC}^{2}+{MCC}^{2}}$$

where $TC$ is the Tanimoto coefficient between drug FP2 fingerprints^63^ and $MCC$ is the similarity of drug mechanism of action defined in Eq. (1). When one of the component metrics, either TC or MCC, is sufficiently high, then the other metric does not need to be as high for the DACS score to be over a predefined threshold. In rare cases of negative MCC values, the MCC component of the DACS score is set to 0.

### Classification datasets

Following the original paper on the AZ-DREAM Challenges dataset^16^, we compiled the primary dataset by excluding those instances having ambiguous synergy scores between − 20 and 20 to create a classification dataset of 3210 drug combinations comprising 2461 synergistic (a synergy score $\ge 20$) and 749 antagonistic (a synergy score $\le -20$) cases. The corresponding augmented dataset contains 1,850,037 synergistic and 465,288 antagonistic combinations totaling 2,315,325 labeled instances. Further, the following four datasets were constructed at varying degrees of drug synergy to evaluate the performance of ML against instances having ambiguous labels, 8817 combinations comprising 5839 synergistic (a synergy score $>0$) and 2978 antagonistic (a synergy score $<0$) cases, 6974 combinations comprising 4882 synergistic (a synergy score $\ge 5$) and 2092 antagonistic (a synergy score $\le -5$) cases, 5408 combinations comprising 3913 synergistic (a synergy score $\ge 10$) and 1495 antagonistic (a synergy score $\le -10$) cases, and 4180 combinations comprising 3119 synergistic (a synergy score $\ge 15$) and 1061 antagonistic (a synergy score $\le -15$) cases.

In addition to the primary dataset, an independent validation set was created based on DrugCombDB^75^. Applying the same synergy score criteria and excluding molecules with the TC of ≥ 0.4 to any compound in the AZ-DREAM Challenges dataset resulted in 250 drug combinations with 14 synergistic and 236 antagonistic effects, referred to as “unseen” data.

### Feature vectors

Input data for machine learning consist of drug and cell features. The former are computed with Mol2vec^81^ by encoding a drug chemical structure to a 300-dimensional vector. The latter features are calculated by embedding 17,419 gene expression values for a cell line obtained from the AZ-DREAM Challenges dataset with an adversarial deconfounding autoencoder^82^. Similar to drug embeddings, the gene expression profile is encoded to a 300-dimensional vector. The final, 900-dimensional feature vector is generated by concatenating two drug feature vectors and a cell feature vector.

### Cross-validation protocols

Two cross-validation procedures are employed utilizing a random and a tissue-based data split. In the random-split cross-validation, the classification dataset is randomly partitioned into five equal-size folds. In the tissue-based cross-validation, the dataset is assigned to five groups according to the tissue type of cell lines, the breast tissue, the digestive system, the excretory system, the respiratory system, and other tissues. Note that tissue types in the augmented dataset are the same as in the original dataset because the augmentation process does not affect cell lines. A fivefold cross-validation is conducted the usual way, i.e., in each round, the machine learning model is trained on the augmented data for 4 subsets and then validated against the original AZ-DREAM Challenges instances in the remaining subset. This protocol ensures that the augmented data is used only to train classifiers and the validation is performed on the original data and labels. Since the original dataset is imbalanced, comprising 76.7% synergistic and 23.3% antagonistic instances, a stratified split is used to preserve the percentage of samples for each class in each fold. When augmenting the training set, the ratio is preserved by proportionally adding instances of each class. In the tissue-based split, although the proportions of synergistic and antagonistic instances are different in each tissue, the training set is augmented in a way to preserve the ratio of synergistic/antagonistic instances in individual folds.

### Machine learning

Four machine learning models are used to evaluate the performance of supervised learning algorithms on the original and the augmented datasets of drug combinations, Logistic Regression, Support Vector Machines, Random Forest, and Gradient Boosting Trees. LR is a supervised machine learning algorithm designed for binary classification tasks to predict the likelihood of an instance belonging to one of two classes (synergistic or antagonistic in our case). It employs the logistic function to transform a linear combination of input features into a probability score, allowing for intuitive interpretation^68,69^. Model training involves minimizing the logistic loss function through optimization techniques such as gradient descent. The coefficients of the linear equation are estimated during the training process to create a predictive model. The following parameters were used in the LR model: L2 penalty, the tolerance for stopping criteria of 0.0001, the inverse of regularization strength of 0.45, the maximum number of iterations of 500, and class weights set to “balanced” to deal with the imbalanced dataset.

SVM is a powerful supervised machine learning algorithm used for classification and regression tasks. In the classification context, it aims to find the optimal hyperplane in the feature space to maximize the margin between data points belonging to different classes^70,71^. SVM is effective in dealing with high-dimensional features and can handle non-linear relationships through the use of kernel functions implicitly mapping the input features into a higher-dimensional space. The following parameters were used in the SVM model: the regularization parameter of 0.42, a linear kernel type, the tolerance for stopping criterion of 0.001, a probability set to true to enable probability estimation, and class weights set to “balanced” to deal with the imbalanced dataset.

The RF classifier utilizes a collection of individual trees built independently to determine the final output by the majority vote^72^. In contrast, the GBT classifier builds trees additively to reduce the bias of the previous tree, and then combines the output of all trees scaled by the learning rate to calculate the final output^73^. Parameters of both classifiers were manually tuned to optimize their classification performance. The following parameters were used in RF: the number of trees in the forest of 300, the minimum number of samples per leaf node of 85, the number of features to consider for the best split equal to the square root of total number of features, and class weights set to: “balanced” in order to deal with the imbalanced dataset. The following parameters were used in GBT: the number of boosting stages of 650, the minimum number of samples per leaf node of 120, the number of features to consider for the best split equal to the square root of total number of features, the learning rate of 0.28, and the maximum depth of the individual regression estimators of 5. In validation calculations against “unseen” data, a GBT model is first trained on the AZ-DREAM Challenges dataset, utilizing either the original instances or the original and augmented data. The trained model is then employed to classify instances in the DrugCombDB dataset^75^.

## Data Availability

All data are freely available at https://github.com/MengLiu90/Synergy-Data-Augmentation.
